# Non-invasive prenatal testing in Germany: a unique ethical and policy landscape

**DOI:** 10.1038/s41431-022-01256-x

**Published:** 2022-12-12

**Authors:** Hilary Bowman-Smart, Claudia Wiesemann, Ruth Horn

**Affiliations:** 1 Ethox Centre, Nuffield Department of Population Health, University of Oxford, Oxford, UK; 2 Monash Bioethics Centre, Monash University, Clayton, VIC Australia; 3 Biomedical Ethics Research Group, Murdoch Children’s Research Institute, Parkville, VIC Australia; 4 Department for Medical Ethics and History of Medicine, Göttingen University Medical Center, Göttingen, Germany; 5 Ethics in Medicine, University of Augsburg, Augsburg, Germany

**Keywords:** Ethics, Genetic testing

## Abstract

Non-invasive prenatal testing (NIPT) has been available commercially in Europe since approximately 2012. Currently, many countries are in the process of integrating NIPT into their publicly funded healthcare systems to screen for chromosomal aneuploidies such as trisomy 21 (Down syndrome), with a variety of implementation models. In 2019, the German Federal Joint Committee (G-BA), which plays a significant role in overseeing healthcare decisions in Germany, recommended that NIPT be reimbursed through public insurance. Following this recommendation, NIPT will be offered on a case-by-case basis, when a pregnant woman, after being counselled, makes an informed decision that the test is necessary in her personal situation. This model differs significantly from many other European countries, where NIPT is being implemented either as a first-tier screening offer available for all pregnancies, or a contingent screen for those with a high probability of foetal aneuploidy (with varying probability cut-offs). In this paper we examine how this unique approach to implementing NIPT in Germany is produced by an ethical and policy landscape resulting from a distinctive cultural and historical context with a significant influence on healthcare decision-making. Due in part to the specific legal and regulatory environment, as well as strong objections from various stakeholders, Germany did not implement NIPT as a first-tier screen. However, as Germany does not currently publicly fund as standard other forms of prenatal aneuploidy screening (such as combined first trimester screening), neither can it be implemented as a screen contingent on specific probability cut-offs. We discuss how German policy reflects the echoes of the past shaping approaches to new biotechnologies, and the implications of this unique model for implementing NIPT in a public healthcare system.

## Introduction

Non-invasive prenatal testing (NIPT) became available in Germany as a publicly reimbursed test in 2022 [1]. In this paper, we describe how a unique ethical and policy landscape has shaped the distinctive implementation model in Germany and discuss the possible implications of this approach. NIPT, a screening test, has become widespread globally since becoming available in Hong Kong in 2011 [2]. It is being implemented into many public healthcare systems to screen for trisomy 21 (Down syndrome, T21), and frequently other conditions such as trisomy 13 (Patau syndrome, T13) and trisomy 18 (Edwards syndrome, T18). There are a range of implementation models; some countries, such as Belgium, have implemented NIPT as a first-tier screen (available regardless of prior probability of foetal aneuploidy). Others, such as France and the United Kingdom (UK), have implemented it as a contingent screen (reimbursed only for pregnancies with a high probability of foetal aneuploidy, as assessed by other methods such as combined first trimester screening (CFTS)) [3].

NIPT uses cell-free foetal DNA in the maternal bloodstream to establish probability of a foetal genetic condition. NIPT is based on a maternal blood sample and is thus considered as ‘non-invasive’, and is generally offered from 10 weeks gestation [4]. NIPT has various features leading to its increasing uptake as a prenatal screening method. It is commonly used to screen for three autosomal aneuploidies —T21, T13 and T18. The performance of NIPT is higher than other prenatal screening tests, such as CFTS; for example, it has a sensitivity of over 99% for T21, although these figures vary for different conditions [3]. However, NIPT results are not diagnostic, and the possibility of false positives remain. Therefore, it is recommended that any high-probability result be confirmed through further diagnostic tests (amniocentesis or chorionic villus sampling—CVS) [4]. Beyond the mentioned aneuploidies, NIPT can screen also for other autosomal aneuploidies, foetal sex, sex chromosome aneuploidies, microdeletions, and various single-gene disorders, although these are not generally publicly funded where NIPT has been implemented in public healthcare systems. In the future, NIPT may be available for any information in the foetal genome [4].

The ethical challenges presented by NIPT are not entirely new. However, NIPT makes these challenges even more pressing. For example, there are concerns that implementation of a more accurate test will result in ‘screening out’ people with conditions such as T21 [5]. Furthermore, there are implications for informed choice, which is of particular importance given that concepts like enhancing ‘informed choice’ are the explicit purpose of prenatal screening programmes in countries such as the UK (although not in Germany, as we will discuss) [6]. Elsewhere, we recently discussed the debate about the concerns and likelihood of NIPT becoming ‘routinised’ as part of standard prenatal care, such as that it may undermine free and informed decisions by the pregnant woman [7].

How these questions are addressed varies between different countries and contexts. Here, we explore the current context in Germany, and how this is affected by unique cultural and historical factors. Germany’s approach to NIPT differs significantly from neighbouring European countries. Rather than implementing NIPT as a first-tier screen, or contingent screen with a probability cut-off, Germany is choosing to offer NIPT on an individual case-by-case basis. We describe the German approach and discuss possible reasons for it, as well as practical and ethical implications.

## Current context of prenatal screening and diagnosis in Germany

### Access to testing

The German healthcare system operates based on health insurance, with the majority of the population being covered by statutory public health insurance. The Federal Joint Committee (*Gemeinsamer Bundesausschuss*, G-BA) decides what medical procedures are covered by the statutory public health insurance, based on what is ‘adequate, appropriate, and economical’ [8].

Unlike many other countries, there is no publicly-funded population prenatal screening programme for chromosomal aneuploidies (including methods such as CFTS). Prenatal aneuploidy tests such as CFTS are available privately but are not covered routinely by public health insurance^1^. As in many other healthcare systems, diagnostic techniques (amniocentesis or CVS), are reimbursed where there are specific indications, such as maternal age (>35) or ultrasound findings (e.g. structural anomalies); ultrasound procedures are publicly reimbursed three times during the pregnancy [9, 10].

NIPT first became available in Germany in 2012 with ‘PraenaTest’, offered by LifeCodexx AG [11]. After this, other commercial tests expanded to the German market, such as Harmony [12]. Until now, those seeking NIPT had to pay privately; the price in Germany has dropped significantly over the past decade, ranging between 200 and 550 € as of 2018 [13, 14]. Exact data on current uptake of NIPT are not available; however, in 2019, the German Board and College of Obstetrics and Gynecology estimated a possible uptake of up to 90% in Germany upon implementation of public reimbursement [15]. While data are limited, there is evidence there is demand; German prospective parents are willing to pay for additional prenatal aneuploidy screening and diagnostic services [16, 17]. In neighbouring countries, such as the Netherlands, publicly subsidised NIPT has been associated with increased uptake for foetal aneuploidy screening, which may have implications for NIPT demand (N.B. in the Netherlands, at this moment in time, NIPT is only partially subsidised and there remains a higher out-of-pocket cost (175€) than in countries such as Belgium) [18].

In 2019, the G-BA recommended NIPT be covered under statutory public health insurance for T21, T13, and T18, available for reimbursement from 2022. As stated, the German approach differs significantly from other countries implementing NIPT, in that NIPT is being offered on an individual case-by-case basis. More specifically, according to the *Mutterschafts-Richtlinien* (Maternity Guidelines, Mu-RL) NIPT can be reimbursed ‘when it is necessary to enable the pregnant woman to deal with her individual situation… within the framework of medical assistance. A statistically increased risk of trisomy alone is not sufficient for the use of this test.’[10] (p.11). In terms of decision-making, the guidelines specify the decision is made by the pregnant woman: ‘The information and advice… serve the goal of an independent informed decision of the pregnant woman.’ [10] (p.12). The standard information brochure for pregnant women appended to the Mu-RL states: ‘This test is not a routine examination. The costs are covered: if other tests have indicated a trisomy, or: if a woman and her doctor decide that the test is necessary in her personal situation. This situation can arise when the possibility of a trisomy burdens a woman so much that she wants it clarified.’ [10] (p.48).

### Regulation of NIPT in Germany

Various laws, regulations and guidelines relate to the implementation of NIPT in Germany. The Genetic Diagnostics Act (*Gendiagnostikgesetz*, §15) regulates genetic testing, aiming to prevent genetic discrimination. This states prenatal genetic testing may only be done for ‘medical purposes’; foetal sex cannot be disclosed until after the 12th week of pregnancy; prenatal screening for adult-onset conditions is not permitted; and a pregnant woman must receive counselling prior to screening or diagnosis [19]. Another law is the Pregnancy Conflict Act (*Schwangerschaftskonfliktgesetz*), which describes the requirement for counselling relating to the prenatal diagnosis prior to termination of pregnancy (TOP) [20]. Section 218 of the criminal code (*Strafgesetzbuch*, §218) outlines criteria for situations where TOP may be permissible. TOP until the 12th week after conception, (14th week of gestation), is permitted with mandatory counselling; after the 12th week, it is permitted if there is ‘danger to life or danger of serious impairment of the physical or mental health of the pregnant woman, that cannot be reasonably averted in any other way’ [21]. There was an exception permitting abortion in the case of foetal anomaly, but this was abolished in 1995 to avoid value judgement about ‘disabled life [22]; Schlegel describes this as an ‘ethical necessity for Germany to avoid any comparison with the Nazi Weltanschauung [worldview]’ [23]. This relates to broader philosophical critiques of selective reproduction such as the ‘expressivist objection’, which argues that selection against disability inherently expresses a negative view of disability [24–26].

Another law relevant to new healthcare technologies (and thus NIPT), is the Social Code (*Sozialgesetzbuch*), which outlines general regulations relating to provision of healthcare in Germany. Book 5 §2 states ‘the quality and effectiveness of the services must correspond to the generally recognised state of medical knowledge and take medical progress into account.’ [27] This can be interpreted as an obligation for the state, or state institutions, to provide NIPT if demonstrated to perform better than other forms of prenatal screening and diagnosis, in addition to being ‘economical’. As previously mentioned, there are also the Maternity Guidelines (Mu-RL), produced by the G-BA. These guidelines regulate provision of medical care during pregnancy and childbirth. The primary goal of the Mu-RL is for ‘early detection of high-risk pregnancies and high-risk births’ [10] (p.2).

## Dominant ethical concerns in Germany

### Concerns about discrimination and the shadows of the past

One of the key ethical concerns in the German debate relates to questions around the ethics of selective reproduction, eugenics, and the (de)valuation of certain lives (e.g. people with T21) [28]. While these are present in other countries, they are particularly emphasised in Germany due to historical context. Of particular concern for a number of stakeholders is that implementing NIPT in the public healthcare system (particularly if it were a population screening programme) could lead to the disappearance of people born with disability, due to fears about possible increases in selective TOP. There is significant criticism on this point across a spectrum of civil society. These include disability rights organisations, such as *Lebenshilfe*. There is also strong engagement by religious organisations, who may also object to TOP in general. The organisation Network Against Selection Through Prenatal Diagnosis criticises prenatal screening and diagnostic technologies from a feminist and disability rights perspective [14].

Arguments around eugenics are strongly coloured by history. Foth has discussed the impact of historical context on German public discourse surrounding NIPT [28]. Foth describes how ensuring that ‘anything that resembled the crimes of the Nazi era’ cannot occur again became an important feature of German policy discourse after World War II, and ‘avoiding selection and eugenics should be regarded as a central part of the German post-war moral identity’ [28]. There is also the view that implementing NIPT (and the assumed follow-on practice of selective TOP) promotes discrimination against people with conditions such as T21 [29]. As one aspect of ameliorating this concern, counselling (prior to test offer and/or prior to TOP) must be balanced and non-discriminatory and inform prospective parent(s) adequately and appropriately about having a child with a particular condition. However, this is challenging in the prenatal context where it is difficult to assess to what extent the child would be affected and possible associated co-morbidities. Information about T21 has been described as ‘either too negative or too optimistic’ [29].

## Framing NIPT: ‘lifestyle’ or ‘medical’ technology

Related to concerns about discrimination is the question of whether NIPT serves a ‘medical’ purpose. Braun and Könninger have described how this debate has evolved in the German context since the introduction of NIPT to the commercial market in 2012 [14]. Multiple actors in civil society, including those mentioned above (e.g. *Lebenshilfe*), have argued that NIPT does not serve a ‘medical’ purpose, as there is no treatment available prenatally for conditions such as T21; rather, it is a ‘selection technology’ that functions as a ‘lifestyle’ product [14]. This distinction is important because the G-BA’s role relates to the regulation and funding of *medical* procedures and diagnostics, and thus to be eligible for public reimbursement, technologies such as NIPT must have a *medical* purpose. The G-BA accepted NIPT as a medical product, and chose not to consider ethical and social aspects of the debate – limiting themselves to ‘technical’ questions—because these were outside their remit [14]. The German Ethics Council, a body with a legal mandate to evaluate ethical issues, produced a 2013 report on the future of genetic diagnosis (including NIPT), but otherwise did not appear to publicly contribute or provide comment for the G-BA decision [30].

Braun and Könninger describe how the manufacturer of PraenaTest, when introducing it to the market in 2012, emphasised the ‘medical’ purpose of NIPT—reducing miscarriage rates by reducing need for invasive testing [14]. The press release stated that PraenaTest will ‘save the lives of up to 700 children per year in Germany’ (note the use of the word ‘children’ here) [14, 31]. pro familia, a German reproductive services and advocacy organisation, also emphasised the role of NIPT in preventing miscarriages when defending the introduction of NIPT [14]. The Federal Ministry of Education and Research provided funding to LifeCodexx for NIPT R&D of ~300,000€; there has been significant criticism of this as endorsing or encouraging NIPT [14, 32]. The framing of NIPT as a 'medical' technology was vital, because this allowed a sidestep from the question of state-supported eugenics. If selective reproduction is explicitly excluded as an aim of NIPT, this validates the G-BA’s positioning of NIPT as a ‘medical’ technology with a ‘medical’ purpose - that is, saving lives (by preventing miscarriages). This means that the policy framework and explicit purpose of implementing NIPT in Germany differs markedly from other countries, such as the UK. This debate also relates to the long-standing broader tension between the ‘reproductive autonomy’ and ‘public health’ rationales for prenatal screening programmes in other contexts [33]. Further below we discuss how, within the German policy and regulatory environment, this framing of NIPT as a ‘medical’ technology that serves a ‘medical’ purpose was essential for the G-BA to make a decision about public funding of NIPT.

### Defending against threats to autonomy presented by new technologies

Germany tends towards restrictive policies for new reproductive technologies [28], and has been described as having a strong ‘techno-skeptical’ camp that cuts across political divides [34]. One of the concerns is that new technologies could undermine individual autonomy, which, in the German context, is often understood as the right to refuse a medical treatment or procedure rather than the right to access it. In other countries, the purpose of NIPT (as well as other prenatal screening and diagnostic technologies) has been described as enhancing or facilitating reproductive autonomy and/or informed choice. For example, the Foetal Anomaly Screening Programme in the UK specifies ‘Services should provide women with high quality information, so they can make a personal informed choice about their screening and pregnancy options.’ [6]. In these other contexts, concepts such as ‘reproductive autonomy’ and 'informed choice' are frequently understood in terms of supporting prospective parent(s) to make the best personal decisions for them, which may require facilitating test access [35].

While concerns about routinisation of NIPT appear widely in the literature [36], the debate is particularly charged in the German context. Research comparing views on NIPT in Germany, Poland and Russia demonstrated a particular focus placed on routinisation in Germany [37]. Similar results were generated by a comparison of the debate in Germany and Israel [38]. Thus, the public funding of NIPT, due to concerns around routinisation, is understood by some participants in the debate as a challenge for individual autonomy, rather than supporting it [38]. The fear is that if NIPT is routinely offered through a publicly funded ‘screening programme’, it can be implicitly perceived as endorsed or recommended by governments and society, and thus women may feel pressured to accept the offer. Hence, in Germany the concern about routinisation and accompanying threats to reproductive autonomy has significantly impacted policy questions around offering NIPT as a ‘screening programme’ [28]. The German discourse on reproductive autonomy focuses more on the ‘right not to know’ and the right to *decline* a prenatal test [35]. The first paragraph of the standard information pamphlet, after the introduction, illustrates this focus: ‘All prenatal examinations are voluntary - this means you can refuse an examination or test that is offered at any time without giving reasons. Your right not to know is so important that no one should pressure you into an investigation.’ [10]. Refusing the implementation of NIPT, as with other novel reprogenetic technologies, can indeed be understood as a form of collective self-determination rather than denial of autonomy [34]. However, while concerns about routinisation are prominent in public debate, research from the Netherlands (within the context of a first-tier NIPT screening programme) found that women did not feel a social pressure to test, or pressure to make a particular decision [7]. Further research in other contexts to provide a more rigorous evidence base for these concerns would be desirable.

Furthermore, in German law, the right to self-determination and the dignity of the pregnant person must be balanced against the human dignity and right to life of the foetus. Indeed, as previously described, in order to access TOP in Germany, the pregnant woman must undergo mandatory counselling. The relevant legislation (*Schwangerschaftskonfliktgesetz* §5; *Strafgesetzbuch* §219) explicitly states that the ‘Counselling serves to protect unborn life’ [39]. Furthermore, the woman must recognise the ‘unborn [child]’ has its own ‘right to life’ and thus TOP should only be considered when continuation of pregnancy would ‘exceed the reasonable limit of sacrifice’ (*Strafgesetzbuch* §219) [40]. Thus, the wording of the legislation around TOP explicitly recognises foetal interests. This provision, as well as the fear of reverting to state supported eugenics, may explain the objection in the public debate to selective TOP.

## How the unique ethical landscape in Germany has shaped policy

The ethical landscape in Germany is indelibly shaped by unique cultural and historical factors, and this contributes to the German policy approach relating to prenatal screening. It is important to distinguish between the conceptual idea of screening as compared to diagnosis, and screening in the sense of a population-level or publicly funded programme actively seeking out conditions. The latter can be described in the German context as ‘*Reihenuntersuchung*’ (mass-screening). In light of the German history of eugenics, there is significant concern in the public discourse around population screening, particularly *genetic* population screening, and the possibility that it could undermine individual choice for the sake of the general population [41]. Klein and Rost describe how the eugenic goals of genetics during the WWII period has since affected conversations around screening in Germany, leading to a critical and cautious approach to any form of genetics-based population screening both in public discourse and in policy [42]. This concern about the public funding of NIPT effectively being a form of ‘*Reihenuntersuchung*’ is expressed, for example, in a 2021 open letter to the G-BA endorsed by 21 different civil society organisations [43]. The G-BA considered this to be a significant enough concern that they directly address it in their list of ‘frequently asked questions’, clearly indicating that the organisation does not want the provision of NIPT to be seen as a form of ‘*Reihenuntersuchung*’, and hence associated with selective reproduction or state-supported eugenics [44]. This is highlighted also in the relevant section of the Mu-RL, with NIPT added under a group of procedures that may only be reimbursed in ‘individual cases…These are not screening tests.’ [10] According to the G-BA, NIPT is dependent on an ‘existing medical or psychological indication’ of the pregnant woman and does not constitute a form of ‘*Reihenuntersuchung*’ within the guidelines on genetic screening.

As noted, many other countries and organisations emphasise concepts such as reproductive autonomy or informed choice as a reason to fund prenatal screening, particularly on a population level. However, Germany is in the unique situation where, within a public healthcare system, prenatal screening methods for conditions such as T21 are not reimbursed as standard. As stated, CFTS is not routinely covered by public funding. There is no existing prenatal screening programme using a genetic test structured around rationales relating to reproductive autonomy, as is the case in other countries. The G-BA decision to add NIPT to the Mu-RL, making it available for public reimbursement, is fundamentally guided by the explicitly stated purpose of the Mu-RL: ‘Medical care during pregnancy and after childbirth is intended to avert possible dangers to the life and health of mother or child and to identify health problems in good time and treat them.’ [10] (p.2). The G-BA notes in their report that there are no curative therapies available for trisomies 21, 13 or 18 [45] (p.12).Thus, the stated justification for publicly funding NIPT must be a ‘medical’ one: specifically, to reduce the number of invasive tests and associated miscarriages. Indeed, NIPT is associated with reduced invasive testing rates elsewhere [46, 47].

This rationale—reducing invasive test rates, and thus potential additional miscarriages—is based on a 2018 technical report by the Institute for Quality and Efficiency in Health Care (IQWiG) [48]. Using an additional miscarriage risk from invasive testing procedures between 0.2 and 1%, the report estimates between 0 and 2 additional miscarriages per 100,000 pregnancies where NIPT is offered as a second-tier screen (1–4 for first-tier screening), compared to up to 47 without NIPT. Miscarriage risk data were drawn from a 2013 report by the German Society for Ultrasound in Medicine [49]. However, at the time of the report, more recent evidence was available (including a 2017 Cochrane systematic review) suggesting the evidence for miscarriage risk associated with invasive procedures was frequently of low quality or imprecise. This Cochrane systematic review was not cited in the IQWiG report, although it had been published approximately two months (September 2017) before the date in their stated search strategy for the Cochrane database - December 2017 [48] p.116) [50]. Furthermore, recent research suggests the risk of additional miscarriage associated with invasive procedures may be much lower than previously thought, possibly even negligible or no additional risk [51–53]. A lower miscarriage risk, however, would weaken the ‘medical’ justification required to reimburse NIPT.

Thus, there are two key facts that appear to be the case. Firstly, a major policy change (publicly reimbursing NIPT) has been developed with a rationale (i.e. a 'medical' purpose of reducing miscarriages) based on what now appears to be weak evidence. Secondly, due to restrictions of various guidelines and legislation, the G-BA only had the option of using such a ‘medical’ justification if NIPT were to be publicly funded. Other factors of this existing situation—such as there being no current publicly funded population prenatal screening programme—also may go some way to possibly explain the approach that Germany has taken. As previously stated, several other European countries, such as the UK and France, have taken a contingent approach, where the provision of NIPT is determined on an established prior probability of foetal aneuploidy through other methods such as CFTS. However, it is of course not possible for Germany to establish a prior probability threshold for public reimbursement of NIPT, because the usual means of establishing the probability threshold (e.g. CFTS) is itself not routinely publicly reimbursed—and thus cannot be considered available to everyone. Furthermore, the section of the Mu-RL where NIPT has been added already specifies that such tests can only be offered on an individual basis. This has led to a scenario where, in Germany, a case-by-case model for NIPT was the only option. By making NIPT available for reimbursement in individual situations where the woman and her doctor ‘have decided together that the test makes sense for her’, the G-BA also seems to emphasise the importance of discussion and critical reflection on the decision to take place prior to taking up the test. Given the described concerns in public discourse around routinisation, this could also be seen as an attempt to avoid routinisation and enable informed decision-making.

However, with this quite generally worded criteria, it is possible that this approach will lead to NIPT being *de facto* available to anyone who wants the test. This could result in the implementation of NIPT, in practice, resembling the first-tier screening model used in countries such as the Netherlands or Belgium. As we have previously noted, concerns about routinisation in these contexts, such as women feeling a pressure to test, do not appear to have come to pass; nonetheless, the importance of counselling and autonomous choice in these contexts is still emphasised [7]. However, if NIPT implementation comes to resemble a first-tier model in Germany, the stated ‘medical’ justification of this approach may be weakened. This is because, at this point in time, although still much higher compared to other types of prenatal screening tests (e.g. CFTS), NIPT in populations with low prior probability of foetal aneuploidy has a lower positive predictive value than when it is used as a contingent test following a high-probability result from other tests such as CFTS (i.e. thus potentially resulting in more false positives). Therefore, if NIPT is sought by many women with low prior probability of a trisomy who previously could not access publicly funded prenatal screening, the case-by-case model may paradoxically result in more invasive tests compared to a straightforward contingent model, rather than less [54–56].

Furthermore, we raise the concern that German legislation surrounding TOP may potentially motivate women who receive a high-probability NIPT result to opt for early TOP and avoid additional waiting time to undergo further confirmatory diagnostic tests, even though the GB-A strictly advised against such a practice. This is because TOP in Germany is easiest to access at the woman’s request up until 12 weeks after conception (14th week of gestation) when conflict counselling through an independent organisation is required, yet no medical or criminal indication is needed. After this period, as previously described, TOP can be accessed under a limited set of circumstances [21, 40]. According to a report by pro familia, women in Germany encounter a range of difficulties in accessing TOP for medical indications (including foetal anomalies), and they estimate that one third of TOPs from week 12 since conception are performed abroad (particularly in the Netherlands) [57]. These factors could potentially push women to prioritise early TOP based on NIPT even if this decision is based on inconclusive or non-diagnostic results. We stress that there is no evidence of this currently occurring; rather, we raise it as a likelier possibility within the German legislative context surrounding TOP provision combined with the timing of NIPT during pregnancy.

Another consideration, potentially positive, is that making NIPT available to women without the use of strict quantifiable criteria may actually enhance the ability to make decisions about pregnancies. If a predetermined probability threshold is a key criterion for accessing NIPT, as is the case in other countries such as the UK, individual and personal preferences of women who do not meet such criteria are not taken into account. Yet, a woman with a low probability of carrying a foetus with T21 (or another trisomy) may nevertheless have other strong reasons to prefer to undergo the test. In the German case-by-case model, her access to NIPT could be secured, if it is suitable based on her ‘personal situation’.

On the other hand, if NIPT were to be implemented as a screening programme with more explicit criteria, this could improve the quality and consistency of healthcare provision and the experience of women. It would further allow a clearer and more robust ethical and scientific debate about what criteria should be used for NIPT provision. The case-by-case model has room for interpretation as to decision-making and what constitutes an ‘unreasonable burden’ for a woman, which may result in unequal offer and access to NIPT. However, to address such situations, the G-BA have produced standardised information provision processes (including a full brochure) and counselling guidelines for NIPT provision [10]. Nonetheless, where room for interpretation exists, concerns about consistency of care and equality of access remain. 

## Conclusion

By implementing NIPT on a case-by-case basis, avoiding any form of prenatal genetic population screening and trying to navigate the regulation and charged social discourse around NIPT, the unique German approach is likely to lead to NIPT being widely available for prospective parent(s). The further implications of this will significantly depend on how NIPT will be presented and offered, and what information will be given. Furthermore, the impact will be shaped by how follow-up procedures, whether it is further prenatal tests or access to TOP, will be managed in clinical practice. Our analysis of the ethical and policy landscape that has shaped this approach and discussion of possible implications, can help inform further debate, as well as considerations for clinical practice and future recommendations.

## Footnotes

1 In practice, it is possible to refer a pregnant woman for CFTS in some cases, in which case the costs can be covered by the health insurance.
